# Emotional distress among adolescents living with perinatal HIV in India: examining predictors and their mediating and moderating effects

**DOI:** 10.1186/s13034-023-00587-x

**Published:** 2023-03-15

**Authors:** Archana Verma, Krishna Kiran Kota, Sampada Bangar, Girish Rahane, Nayana Yenbhar, Seema Sahay

**Affiliations:** 1 Division of Social and Behavioral Research Sciences, ICMR-National AIDS Research Institute, 73, G-Block, MIDC, BIE, Pune, 411026 India; 2 Department of Anthropology, Savitribai Phule Pune University, Ganeshkhind, Pune, India; 3 ICMR fellow, Division of Social and Behavioral Sciences, ICMR-National AIDS Research Institute, Pune, India; 4 Department of Health Promotion & Behavior, School of Public Health, Georgia State University, Atlanta, USA; 5 Division of Social and Behavioral Sciences, ICMR-NARI, Pune, India; 6 Division of Epidemiology, ICMR-National AIDS Research Institute, Pune, India

**Keywords:** HIV, Adolescents living with perinatally acquired HIV (ALPHIV), Anger, Mental health, Career goals, Sexual relations, Parents, Emotional distress, India

## Abstract

**Background:**

Development of emotional distress (ED) among adolescents living with perinatally acquired HIV (ALPHIV) affects their adherence behaviour and social and psychological functioning. Data on stressors among ALPHIV demonstrates the gap on the predictors of ED experienced by ‘perinatally infected ALHIV’ in the Indian socio-cultural milieu. This study aimed to identify the predictors of ED and examine their mediating and moderating role in the development of ED among Indian ALPHIV.

**Methods:**

Utilizing the sequential exploratory mixed-methods design, 43 qualitative interviews were conducted with ALPHIV, parents/guardians, and health care providers, followed by the cross-sectional survey among 100 ALPHIV (10–19 years). The distress subscale of the Weinberger Adjustment Inventory was used to measure ED. Qualitative data, analyzed using grounded theory were utilized to develop a survey tool. Bivariate and regression analyses were conducted to identify predictors of ED. Mediation and moderation models were tested to examine underlying mechanisms associated with ED. The study was approved by the institutional ethics committee.

**Results:**

Strong parental control, compulsive asexuality, perceived negatively different from peers, and anger toward parents were the major themes emerging from the qualitative component which eventually led to survey tool domains viz., HIV awareness, parental control, hypervigilance, adolescent-parent relationship, adolescent-parent communication, body image and perceived negatively different from peers (PNDP). Quantitative analysis indicated high ED among ALPHIV and ED was significantly associated with PNDP, anger, body image, and hypervigilance. The relationship between PNDP and ED was significantly mediated by anger, and moderated by body image and hypervigilance.

**Conclusions:**

The study stresses the need for early mental health interventions for Indian ALPHIV before an ALPHIV develops ED. Focused counseling on anger assessment, body image issues, and self-perception is critical for leading a 'normal' life by ALPHIV. Besides, skill building of primary caregivers is recommended to draw a line between protection and overprotection.

## Background

Adolescents (10–19 years of age) are known to experience stress and mood swings which result in an increased risk of violence, aggression, substance abuse, accidents, and risky behaviors. The presence of chronic illnesses such as HIV increases the risk of psychosocial problems among adolescents [1–3]. Successful national antiretroviral therapy (ART) rollout program has increased longevity and hence, HIV-infected children are able to reach adolescence [4, 5]. The additional issue emerges as chronic HIV disease poses not only biological but also psychosocial challenges among Adolescents Living with HIV (ALHIV). They usually experience higher stress than their uninfected counterparts because of their health condition, loss of parent/s, missing recreational activities, having to take daily medication, and many a times face side-effects, stigma, and discrimination [6, 7]. Studies have shown that there is a range of mental health problems prevalent among ALHIV such as anxiety, depression, hyperactivity, conduct disorders, and emotional distress [6, 8–10]. Anger, body image, and self-acceptance issues predispose ALHIV to emotional distress (ED) which is found to be associated with negative life events among them [6, 11]. Anger is directed towards family, friends, school, the presence of HIV and medication, and it was high among HIV-infected children [12]. Having a chronic illness due to the circumstances that are beyond adolescents' control, leads them either to become irresponsible and rebellious towards caregivers whom they blame or believe to be responsible for their illness or may withdraw into depression and isolation [13]. Physiologically, lipodystrophy syndrome, stunted growth, and delayed bodily development among ALHIV were shown to lead to ED and social isolation [14].

Most often ED remains undetected because it gets masked by the physical complaints which are explained as “understandable” distress of the disease [15, 16]. Studies reflect the existing gaps in understanding and factors affecting emotional distress among children and ALHIV [17–19]. The present study aimed to identify the predictors of ED among adolescents living with perinatally acquired HIV (ALPHIV) and to understand the underlying mechanisms leading to ED by exploring the potential mediating and moderating roles of significant correlates.

## Methods

### Study design

During 2014–17, using sequential exploratory mixed-methods design, this study was conducted in two phases: (1) Qualitative phase, and (2) Quantitative phase.

### Qualitative Phase

This phase was an exploration and examination of stressors leading to ED among ALPHIV. The critical realists paradigm and grounded theory approach were used to collect and analyze the data [20–23]. Data were collected from parents/guardians, adolescents including ALPHIV and HIV-uninfected adolescents, and health care providers to understand the stressors which could lead to ED among ALPHIV.

- ***Sample size***: Qualitative interviews were conducted to explore the psychosocial issues of ALPHIV. A total of 43 qualitative interviews were conducted with key stakeholders which included 23 in-depth interviews (IDI) and 20 key informant interviews (KII). The stakeholders comprised of: 1) primary caregivers (parents/guardians) of adolescents (n = 12), 2) adolescents (n = 11), and 3) Healthcare providers (n = 20) that included paediatricians, clinicians, mental health experts, social workers, public health researchers and program personnel from private, government, and non-government organizations. Of the 11 adolescents, seven were ALPHIV and four  were HIV-uninfected adolescents.
- ***Participant recruitment and data collection***: Using purposive and convenience sampling techniques, interviews were conducted with consenting participants identified from different geographical regions in India viz. Delhi & Lucknow (North India), Chennai & Puducherry (South India), and Mumbai, Pune & Goa (West India). Parents/guardians, ALPHIV, and HIV-uninfected adolescents were identified from ART Centres, government and non-government organizations (NGOs), and the community. Primary caregivers and ALPHIV were also approached through meetings and support groups of people living with HIV & AIDS (PLHIV), community involvement programs, and clinical settings of ICMR-National AIDS Research Institute (ICMR-NARI), Pune. Healthcare providers (HCPs) were identified based on their expertise in paediatric and adolescent HIV management, adolescent health, mental health, and national health programs. HCPs were approached through emails/phone calls and prior appointments were taken. The snowball sampling technique was also utilized to identify the participants. The purpose of the study was explained to each potential participant and willing participants were interviewed. Eight HCPs expressed their inability to participate due to lack of experience in the required field and one ALPHIV could not participate because of parent’s objection. Therefore, more potential participants were identified and approached in the same categories to reach data saturation. Interviews were conducted face-to-face and audio-recorded. Three interviews were documented using field notes as permission for audio-recording was denied. The qualitative interviews were conducted by a masters-level trained anthropologist till data saturation was achieved.
- ***Focus of the qualitative tools***: Qualitative interviews were conducted using interview guides, focused on the adolescents' developmental issues, psychosocial health issues, and diverse needs of ALPHIV. Barriers and facilitators in the management of ALPHIV were also explored. Separate guides were developed for parents/guardians, HCPs, and adolescent categories respectively. The interview guides were pilot tested to check the flow of topics, comprehension of probes, missing probes, or need of rephrasing, and accordingly interview guides were modified.
- ***Qualitative data analysis***: The interviews were transcribed verbatim, translated into the English language as applicable, and typed in Microsoft Word. The qualitative data were analyzed in QSR NUD*IST version 6.0. Initially, 3–4 transcripts were read to break, examine and conceptualize the categories. Three-step coding guidelines were followed by the researchers (AV & SS) for coding the data [24]. Using grounded theory principles, a priori codes were developed deductively from interview guides and open codes inductively from the data [22].

Following the constant comparison method, linkages between the codes were identified and similar codes were merged into categories. The core categories which validated the relationships between codes or categories were identified and developed into themes. Finally, a thematic analysis was performed.

### Quantitative phase

- ***Sample size and participant recruitment***: A cross-sectional survey was conducted among 100 ALPHIV (10–19 years) from the ART centre and Integrated Counseling and Testing centre (ICTC) in Pune (India). Each consecutive ALPHIV visiting the ART centre/ICTC was screened and enrolled. The perinatal HIV infection was confirmed from parent/guardian or health records. ALPHIV who lived in care homes/orphanages acquired HIV infection through blood transfusion or behaviorally, and reported the presence of any psychiatric problem were excluded. The purpose and procedure of the study were explained first to the parent/guardian and after obtaining the parent’s/guardian’s consent to permit their adolescent ward to participate; ALPHIV was informed about the study. Only the willing and consenting or assenting ALPHIV participants were screened and enrolled in the survey.
- ***Data collection***: The survey data were collected by a trained researcher using a semi-structured questionnaire in a face-to-face interview. The interviews were conducted in a private setting and participants were assured of their privacy and confidentiality. At the end of the interview, a debriefing was done for every participant where their doubts or misconceptions were clarified.
- ***Focus of the survey instrument***: Qualitative data informed the content of the survey instrument. Appropriate domains were designated to the items after multiple discussions among the researchers from the fields of anthropology, public health, and psychology. The survey instrument was composed of 142 items and focused on demographic characteristics along with the following domains which were identified through the qualitative themes/codes:
- *Awareness about HIV status *was measured by three variables: Do you consider yourself ill? Do you have any chronic illness? Are you taking any medications? The response options were “yes”/“no” with detailed specifications such as ‘specify the illness if have any chronic illness and specify the medicines’.
- *Anger*, a 15-items domain assessed the feelings of anger among ALPHIV about illness-specific issues. Items pertained to angry feelings due to disease acquisition; God gave the disease only to me; unaware of the source of infection; a continuous reminder of being taken ‘care of’ while playing with cousins; people expect more work as you are dependent on them; you do more work than children of your age in the family; feel angry like picking up and throwing things or shout on someone; feel like hurting self or banging head; frequency of losing temper; venting out anger on the person from whom you feel you got the disease; and venting out anger on parent/s. Inter-item reliability was good as indicated by the Cronbach alpha score of 0.798.
- *Adolescent-parent relationships (APR)* comprised of 6 items that measured: happiness/satisfaction in one’s relations with parents/guardians. Inter-item reliability was indicated by a Cronbach alpha score of 0.744.
- *Adolescent-parent communication (APC)* consisted of 8 items. It measured the feelings of ALPHIV pertaining to their ability to communicate and share with parents/guardians. The items focused on adolescents' ability to discuss with their father/mother/guardian about friends, future plans, health issues especially sexual health, and relationships with the opposite sex. Inter-item reliability was indicated by a Cronbach alpha score of 0.811.
- *Parental control (PC)* was a 4-item domain to measure the feelings of ALPHIV pertaining to restrictions put by parents/guardians for social activities, family functions, or entertainment with peers. The inter-item reliability was indicated by a Cronbach alpha score of 0.845.
- *Hypervigilance* measured the overt caring behavior of parents/guardian in order to hide the HIV infection. This construct comprised of 4-items: How often are you bothered by your parent/guardian/relatives: (1) stopping you from doing something because of your sickness/ill health, (2) putting more restrictions on you than other children, (3) repeatedly asking to take care of health and medicines, and (4) stopping you from doing something. Inter-item reliability was indicated by a Cronbach alpha score of 0.783.
- *Body image* measured the feelings of ALPHIV about physical looks and pubertal development. This domain consisted of 6 items: How often ALPHIV is bothered by comparing physique with peers or other adolescents, teased by friends for physical looks or growth, self-critical of own physical looks, delayed bodily changes, delayed puberty, and shyness/hesitation for physical looks. Inter-item reliability was indicated by a Cronbach alpha score of 0.774.
- *Perceived Negatively Different from Peers (PNDP)* was the independent variable and measured how the ALPHIV perceived themselves as “different” from their peers in the context of having HIV or chronic illness. It was a dichotomous variable. The question was: Do you feel separate or different from your friends or people of your age? The response was ‘Yes’ or ‘No’ with an explanation for their feelings.
- *Emotional distress (ED)* was adapted from the distress subscale of the Weinberger Adjustment Inventory [25]. The scale has been used among healthy as well as adolescents having chronic illness [26, 27]. The distress dimension of this inventory consists of anxiety, depression, low self-esteem, and low well-being. The distress subscale with 12 items was used in the study. Inter-item reliability was indicated by a Cronbach alpha score of 0.740.

The responses for anger, APR, APC, PC, hypervigilance, body image, and ED were recorded on a 5-point Likertscale. The survey instrument was translated into the local languages (Hindi & Marathi) and back-translated to check the exact meaning of the items in the local language. The tool was pilot tested among 10 adolescents.

d) ***Quantitative data analysis***: The quantitative data were analyzed using Statistical Package for Social Sciences (SPSS), version 25.0. Bivariate analysis was performed to estimate the association of demographics and other study variables with ED. Wilcoxon rank-sum test was used for categorical variables (sex, education status, living arrangements, awareness of HIV status, and awareness of having a chronic illness). Spearman’s correlation was computed for continuous variables such as age and psychosocial measures. Sex, awareness of HIV status, and awareness of having a chronic illness were determined as control variables based on prior evidence. To estimate the conditional associations of the study variables and ED, a multivariable linear regression model was executed with all the study variables and control variables. Mediation and moderation analyses were performed to examine the underlying mechanisms and to identify the potential mediators and moderators that lead to ED among ALPHIV. The point estimates for the path a, path b, path c’, and path c were generated. Bootstrapping (N = 5000) to construct confidence intervals for the indirect effect (path ab) was conducted to determine the statistically significant mediators. The conditional effects of the independent variable on the outcome variable at three different levels of moderator, -1 SD, Mean, and + 1 SD were estimated. PROCESS macros v3.0 by Andrew F. Hayes [28] was used to test mediation and moderation models.

### Ethics

The study was approved by the institutional ethics committee of ICMR- National AIDS Research Institute, Pune (India). Written informed consents were obtained from all the participants prior to their participation in the study. In the case of minor adolescents (less than 18 years), written assent from adolescents and informed consent from their parent/guardian was obtained.

## Results

Four major themes that emerged from the qualitative data were: 1) Strong parental control, 2) Compulsive asexuality, 3) Perceived negatively different from peers, and 4) Angry ALPHIV.

### Strong parental control

Caring of a child having a chronic, life-threatening, and socially stigmatized illness was a challenge for the parents/guardians, inciting fear which provoked overprotective and hypervigilant parenting practices, and over-controlling behaviors among the parents. Both the ALPHIV and the parents/guardians reported similar controlling behavior towards their HIV-positive wards such as putting restrictions on peers, school trips, and other social activities. These were also endorsed by the health care providers.

*“My mother does not allow me to go with friends as she is scared because ART is going on.”* (AD-03, ALPHIV)

A mother shared, *“I do not send her [/daughter/] anywhere because if someone would do bad behavior with us [/stigmatize us/]. Whenever [she] has to go somewhere, anywhere like has to go on a trip, I go with her. I do arrangements like this so that I stay with her.”* (CG-08, Mother).

*“Only thing is there has been a lot of over protection from the parents where they are worried… anxiety is the reason for the overprotection by the parents.”* (HCP-18, Adolescent physician).

It has been found that parents/guardians become so hypervigilant that they tried to keep an eye on the activities of adolescents because of the inherent stigma associated with HIV.

*“She [/daughter/] does not go anywhere alone I always accompany her… No, I do not send her… I always accompany her wherever I take her… do not send her [/daughter/] alone.”* (CG-10, Mother).

Similarly, a guardian of ALPHIV shared: *“We send him = XXX = [/brother’s name/] or uncle with her [/ALPHIV/]… to see what she is doing at friend’s place. She [/grand-daughter/] is not allowed to go alone”.* (CG-04, Guardian).

The controlling behavior of parents/guardians emerged in the need to maintain secrecy about HIV diagnosis to protect their wards from stigma and discrimination. This hypervigilant behavior of parents/guardians seemed to result in captive isolation of ALPHIV.

### Compulsive asexuality

HCPs explained that sexuality is one of the crucial elements of adolescent development and ALPHIV is no exception.

*“It’s a normal phenomenon.* *Getting involved in sexual relations during teenage*. Because the adolescence is the phase to explore and experiment”. (HCP-16, Mental Health Expert).

*“This [/sexuality/] would be [same]. They [/ALPHIV/] do the same as any other adolescent. It is not related to HIV, it [/sexuality/] would be same [/for all adolescents irrespective of HIV/].”* (HCP-08, Programme Personnel).

The parents/guardians not only controlled ALPHIV's social activities but also seemed to control their sexuality and intimate relations.

*“See, your father had done such and such [/multiple sexual relations/]… So this [/HIV/] happened because of going outside [/by keeping sexual relations outside/]. So you do not do this mistake.”* (CG-03, Guardian).

One of the ALPHIV also shared that her mother controls the intimate relationships.

*“Mother has warned me before only that if she comes to know anything about this [/about having boyfriend/], she will not talk to me. I am scared about this”* (AD-01, ALPHIV).

Another ALPHIV shared her worries on marriage and stress of unfulfilled dream.

*“I am crazy about my marriage from my childhood. Means marriage is my dream. I want to do this and that is my marriage. Now [/after knowing about HIV/] my marriage will not happen…Only I was feeling that I will not get married now.” *(AD11, ALPHIV).

The uninfected counterparts of ALPHIV also had strong views about an ALPHIV thinking of having intimate sexual relationships. Stigma seemed to emerge. On asking what would be their reaction if an ALPHIV approached him/her for intimate relationships; the adolescent shared she would admonish as follows:

“I would have told her [/HIV positive friend/] 'No' [/for intimate relationships/], and tell her not to make a physical relationship like this [/in presence of HIV/]”. (AD-07, HIV-uninfected adolescent).

*“There are chances of others getting infected then. The person with whom the infected person marries will get infected. And then when married, they will have kids as well and then they [/children/] will be infected too. That’s the reason they shouldn’t marry”.* (AD-09, HIV-uninfected adolescent).

Any reference to safe sexual behavior was missing. ALPHIV pointed out the gap in counseling sessions on safe sexual practices.

*“They [/ART counsellor/] did not talk about precautions [/for safe sex/], just told how it [/HIV/] spreads and in that only told about sexual relations [/as one of the modes of HIV transmission/]”* (AD-12, ALPHIV).

Some parents/guardians of ALPHIV tended to communicate on developmental changes, especially, reproductive and sexual health (RSH) during adolescence with their ward to caution them about transmitting the infection to others. However, communications about RSH are not initiated with uninfected adolescents. A parent of an ALPHIV son and uninfected daughter responded:

*“We have this [/HIV/] but it should not happen to others. So if a girl is normal and you make relations with her, then it is wrong. I told my son [/about not having sexual relations/], not my daughter.”* (CG-07, Mother).

It is only to keep the ALPHIV asexual or to abstain from sexual exploration, it was evident that parents/guardians of ALPHIV communicated with them about sexual relations. However, such communications in general are avoided and considered taboo in Indian society.

*“A larger number of parents-they maintain a distance with their adolescent children. Sometimes they do not feel like answering their queries, [as] it might be related to a sensitive issue.” *(HCP-16, Mental Health Expert).

### Perceived negatively different from peers (PNDP)

ALPHIV felt that they were different from their peers. Developmental delay, fear of being identified as sick or HIV positive and captive isolation by parents/guardians instilled fear of losing peers and the possibility of rejection from peers resulting in negative self-perception. Frustration, isolation, and self-stigma were voiced by the ALPHIV in this study.

*“Why only I have this disease? Why do you [/uninfected sister/] not have it as well?”* (AD-03, ALPHIV).

An HCP brought this feeling of being different in a negative sense very eloquently: “Diseases which are long term in their course, which has an impact not only on their [/adolescents'/] day to day life but also, on their functioning. So, because of this, many of these [/HIV infected/] children might see themselves as little different from their peers or siblings.” (HCP-07, Mental Health Expert).

Another HCP echoed the same: *“There is this peer pressure. So they do not want to be different from the crowd. Therefore, they do not want to be seen taking medicines by their friends.”* (HCP-20, Paediatrician).

HCPs concurred with the voices of ALPHIV and explained that the impact of disease on their daily routine and peer pressure brought the feeling of being ‘different’.

*“Adolescents, otherwise, is a very healthy phase of life, and therefore, if you are sick, having to take medicines; you are going to always feel that you are different from all other peers and that might affect your self-esteem, and affect your sense of self.”* (HCP-19, Mental Health Expert).

HCPs emphasized that peers are critical for adolescents' social development and that is why fear and stigma of being identified as HIV positive exists strongly among ALPHIV. This impacts their psychosocial functioning and results in social withdrawal and anger.

### Angry ALPHIV

Adolescence is a phase of knowing about the unknown. Of course, ALPHIV become curious about their illness, medications, and their impact on their future aspirations. The following subtheme ‘*blaming/questioning parents' behavior*’ reflects anger against parents.

- *Blaming/questioning parents' behavior*: Once ALPHIV understands the modes of HIV acquisition, especially the sexual transmission mode; it brings curiosity into the mind of adolescents which raises suspicion about the parents' behavior. A mother shared the questions asked by her son:

“*Mother, this HIV I got from you or because of father? You have this [/HIV/], means did you get it from father or outside?*” (CG-07, Mother)

Adolescents start blaming their parents, especially their father as the source of illness in the family. This affects their relationship with their parents.

“*I came to know that it [/HIV/] came through him [/father/] to me. Then, inside, I thought why he did it*?” (AD-01, ALPHIV)

“*About my parents… I was too much angry with my father. Because of him [/father/], mother got [HIV] and they both went [/died/]. But I feel that if my father had not got it [/HIV/] then my mother also would be alive and I also would have been ok. Why would I have to take these two tablets?*” (AD-11, ALPHIV)

Key informants tried to explain how an ALPHIV processes the information about HIV transmission:

“*Given the huge stigma around HIV, if an adolescent comes to know that s/he is infected because of his parents, the very first issue that s/he would face is to deal with the information that why did my parents get infected? Why not the other’s parents? So, is there anything wrong with my lineage itself, and who was wrong? My mother was wrong or my father was wrong? And it all depends on where it comes from and at what stage it is [/how disclosure was done/]. He might feel that others and peer groups will feel that he comes from parents who are not 'morally correct'. That’s the shaming and blaming…”* (HCP-12, Social scientist)

*“This is the basic problem when at that [/adolescence/] age if they [/adolescents/] get to know their [HIV] status; they [/adolescents/] always know now something about sex. So every time they know that their parents have done some ‘bad activity’; that is why they [/parents/] got [HIV] and we [/adolescent/] got [HIV] because of that.”*(HCP-03, Program Personnel)

HIV impacts not only the adolescent-parent relationships; it also affected their future aspirations viz. academics, peer relations, intimate relations, and marriage. This reflects in another subtheme '*Unfulfilled aspirations*'.

- *Unfulfilled aspirations:* Side-effects of ART and frequent hospital visits have an impact on their education. The worry to achieve career goals results in anger.

*“There is too much effect on my studies so I get angry, irritated. That's why I have started this [/ART/]. It also scares me that what if I will not be able to achieve my goal.”* (AD-01, ALPHIV)

The interruptions in education are common as shared by an HCP:

“*A lot of children leave school because of issues related to stigma and discrimination. That is the first impact. Number 2 impact is a lot of times especially before they [/ALPHIV/] start ART and in the initial time they deteriorate despite taking medicine, school absenteeism because of repeated illnesses [/opportunistic infections/]… not only missing school if they just don’t have the energy how do they study. The third impact could be just related to their [/HIV acquisition through parents/]… because perinatal HIV impacts upon the mental development of a child*.” (HCP-11, Paediatrician)

“*They [/ALPHIV/] are not able to go to school regularly when they have to come to the hospital frequently…They are very intelligent children but they are not able to spend that much time on their studies.*” (HCP-10, Mental Health Expert)

When adolescents start understanding HIV as a sexually transmitted disease, they worry about their marriage and the transmission of their infection to their offspring. Visualizing unfulfilled dreams and fear of secondary transmission tended to provoke angry feelings among ALPHIV.

*“I was very much angry on father. But now in my marriage, it [/HIV/] is a barrier and I have only the tension of this thing.”* (AD-11, ALPHIV)

A mother shared the conversation that she had with her HIV-infected son:

*“My son became very angry and said because of father, you got HIV and I also got this. Now my children will also get this [/from me/].”* (CG-07, Mother)

The HCPs agreed with the emotions of ALPHIV pertaining to their sexuality.

*“In adolescents, sexuality is a very key element of identity and if that issue of sexuality is going to be inevitably influenced by the fact that you have a disease that can be transmitted sexually, how that impacts on your mental health particularly.”* (HCP-19, Mental Health Expert)

The dilemma, fear, concerns, and anger led to a critical need for counseling aptly indicated by an HCP as follows:

“*I think they [/ALPHIV/] also need to be counseled about that they can have a normal life, they can get married; they can have children who can be negative. So they can be absolutely normal individuals like anyone else in the society.*” (HCP-20, Paediatrician)

Using these evidences from qualitative research, a survey instrument was developed to conduct a cross-sectional survey to understand the relationship between emotional distress and other psychosocial factors among ALPHIV.

### Profile of quantitative survey participants

A total of 117 ALPHIV were screened, eligible 100 ALPHIV participated in the survey. The mean age of adolescents was 14.95 years (2.57 SD), 18% had dropped out of school, 67% lived in a nuclear family, and 24% considered themselves ill; 69% were aware of having a chronic illness, 52% were aware of their HIV infected status, and 17% had negative perception; hence categorized as PNDP. The mean score for ED was 23.38 (7.81 SD). Table 1 presents the mean scores of psychosocial domains. In the bivariate analysis, PNDP, body image, anger, APR, hypervigilance, and parental control were significantly associated with ED (Table 1).

**Table 1:** Participant demographics and correlates of emotional distress among adolescents living with perinatally acquired HIV (ALPHIV)

Characteristic	N = 100	Test statistic (p-value)
Age, Mean (SD)	14.95 (2.57)	0.12 (0.23)^a^
Sex		
Boy	60	1.43 (0.15)^b^
Girl	40	
Education		
Enrolled	82	0.995 (0.32)^b^
Drop-out	18	
Living arrangements		1.78 (0.41)^c^
Nuclear family	67	
Joint family	17	
Others (with guardians)	16	
Feeling of having sickness		
Yes	24	1.39 (0.17)^b^
No	76	
Awareness of having a chronic illness		
Yes	69	1.67 (0.09)^b^
No	31	
Awareness of HIV status		
Yes	52	0.23 (0.82)^b^
No	48	
ART Use		
Yes	87	− 1.82 (0.07)^b^
No	13	
Perceived different from peers		
Yes	17	2.76 (0.006)^b^
No	83	
Study variables	Mean (SD)	Test-statistic (p-value)
Body Image	11.44 (4.73)	0.46 (< 0.0001)^a^
Anger	26 (8.61)	0.49 (< 0.0001)^a^
Adolescent parent communication (APC)	15.93 (6.77)	− 0.03 (0.77)^a^
Adolescent parent relationship (APR)	20.65 (5.70)	− 0.24 (0.01)^a^
Hypervigilance	7.70 (3.96)	0.47 (< 0.0001)^a^
Parental Control (PC)	7.71 (4.31)	0.35 (< 0.0001)^a^

*a* Spearman’s correlation, *b* Wilcoxon Rank Sum, *c* Kruskal Wallis test, *SD* Standard Deviation

In the multivariable analysis (Table 2), PNDP predicted an increase of 0.19 standard deviations in ED compared to the adolescents who did not perceive themselves as different from their peers.

**Table 2:** Association between emotional distress (ED) and correlates using multivariable analysis

Variable	Unstandardized coefficient (B)	Standard error	95% CI	Standardized estimate (β)	p-value
Intercept	10.74	3.60	3.60–17.90		0.004*
PNDP	3.91	1.75	0.43–7.40	0.19	0.03*
Anger	0.22	0.09	0.04–0.41	0.25	0.02*
Body Image	0.37	0.16	0.06–0.68	0.23	0.02*
APR	− 0.15	0.13	− 0.41–0.12	− 0.11	0.27
APC	0.09	0.11	− 0.13–0.31	0.08	0.41
Hypervigilance	0.57	0.20	0.17–0.96	0.30	0.006*
PC	− 0.11	0.17	− 0.45–0.22	− 0.6	0.51
Sex	0.26	1.24	− 2.20–2.72	0.02	0.834
Awareness of HIV status	− 1.30	1.50	− 4.30–1.70	− 0.08	0.40
Awareness of chronic illness	0.83	1.63	− 2.40–4.06	0.51	0.61

*CI* Confidence Interval, *p-value < 0.05

The relation between PNDP and ED was evident in the qualitative phase as an ALPHIV shared: “*Sometimes it feels like we are different from others because others do not accept us freely…If we can share about this with somebody, then they may not be [/friends/]with us*.” (AD-12, ALPHIV).

Anger and ED coexisted as an HCP narrated about his ALPHIV patient who shouted at his parent saying: “*I got my disease because of you [/parent/]”.* In response, the HCP shared with the interviewer*:“Because they [/ALPHIV/] start getting to know how HIV has happened?*” (HCP-09, Paediatrician).

In the multivariable analysis, this fact was confirmed. Experiencing anger was associated with ED, meaning an increase of one unit in ‘anger’ was predicted to be an increase of 0.25 SD in ED. Additionally, body image and hypervigilance were significantly associated with ED. A mental health expert opined that parents’ hypervigilance could translate into mental health issues among ALPHIV:

*“For HIV even if parents are supportive, they themselves have fear about the larger societal acceptance because of which that [/fear/] might get translated in their reactions to the child… it might develop into some kind of behavioral problems or depressive or anxious personality.”* (HCP-07, Mental Health Expert).

Informed by the theoretical evidence [29–31], qualitative findings from our study, and factors identified in regression analyses, we explored separate mediation and moderation models to assess whether anger, body image, and hypervigilance explained the relationship between PNDP and ED.

PNDP had a statistically significant effect on anger (*path a*). The effect of anger on ED was also statistically significant (*path b)* (Table 3). The indirect effect (*path ab*) of anger was found to be statistically significant on the relationship between PNDP and ED. The total effect (*path c*) of PNDP on ED was also statistically significant when anger was tested as a mediator. The ALPHIV did demonstrate their distress and anger because of their body image in the qualitative interviews as follows:

*“He [/son/] tells about his height as his height is less. So, he asks always what did you eat when [uninfected] sister was in your stomach and what did you eat when I was in your stomach? So he tells me that I had not taken proper care at his time [/during pregnancy while when daughter was conceived she had taken proper care/]”*. (CG-07, mother).

**Table 3:** Total, direct, and indirect effects of psychosocial factors on the relationship between PNDP and emotional distress using mediating effect analysis

Mediator	Path a, SE, 95% CI	Path b, SE, 95% CI	Path c’, SE, 95% CI	Path ab, SE, 95% CI
Anger	6.90*, 2.16, 2.60–11.19	0.44*, 0.08, 0.28–0.61	4.10*, 1.82, 0.49–7.70	3.06*, 1.35, 0.61–5.87
Body image	4.90*, 1.21, 1.67–6.50	0.74*, 0.14, 0.45–1.03	4.10*, 1.85, 0.42–7.79	3.04, 1.27, 0.73–5.73
Hypervigilance	0.19, 1.07, − 1.93–2.32	0.95, 0.16, 0.62–1.27	6.97*, 1.69, 3.61–10.32	0.18, 1.26, − 2.05–2.95

Models were adjusted for sex, awareness of HIV status, and awareness of having a chronic illness. *P < 0.05, SE Standard Error, 95% CI – 95% Confidence intervals

**Fig. 1:**
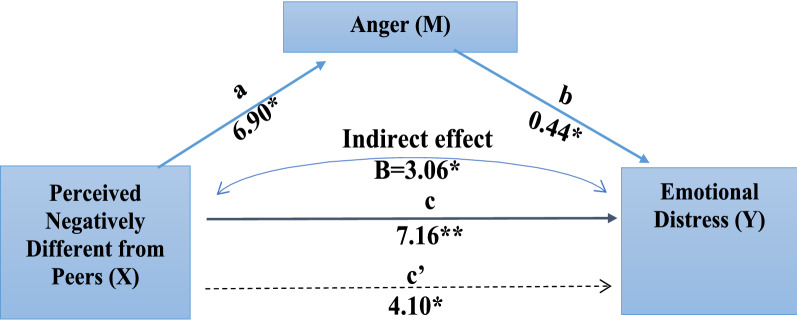
Mediating Effect of Anger on Emotional Distress

Figure 1 shows the pathways explaining the role of anger as a mediator between PNDP and ED.

Subsequently, body image and hypervigilance were tested for the potential role of mediator. In the model testing body image as a mediator, a statistically significant mediating role between PNDP and ED emerged. However, as PNDP is not causally antecedent to body image, a condition that is required to consider a variable to be a mediator [32]. Therefore, the role of body image as a mediator was rejected. For the model testing hypervigilance as a mediator, the indirect effect (*path ab*) on the relationship between PNDP and ED was not statistically significant.

ALPHIV seem to be traumatized about their bodies. Most of them were dissatisfied and worried as seen in the following excerpt:

*“People at home used to say that I looked very much like a bamboo stick, dresses do not look good on me.”* (AD-11, ALPHIV).

In the moderation analyses too, body image was identified as a significant moderator for the relationship between PNDP and ED as the product term (PNDP x body image, XM_1_, *Path c*) was statistically significant (Table 4).

**Table 4:** Moderating effect analysis of psychosocial factors on the relationship between PNDP and emotional distress

Moderator	Path a, SE, 95% CI	Path b, SE, 95% CI	Path ab, SE, 95% CI
Anger	− 5.73*, 6.07, 0.34–− 17.81	0.36*, 0.09, 0.17–0.54	4.10*, 1.82, 0.49–7.70
Body image	− 5.80, 4.87, − 15.44–3.90	0.55*, 0.17, 0.21–0.89	0.71*, 0.32, 0.06–1.36
Hypervigilance	0.67, 3.39, − 6.06–7.40	0.74*, 0.19, 0.37–1.11	0.78*, 0.37, 0.05–1.51

Models were adjusted for sex, awareness of HIV status, and awareness of having a chronic illness. *P < 0.05, SE – Standard Error, 95% CI – 95% Confidence intervals

**Fig. 2:**
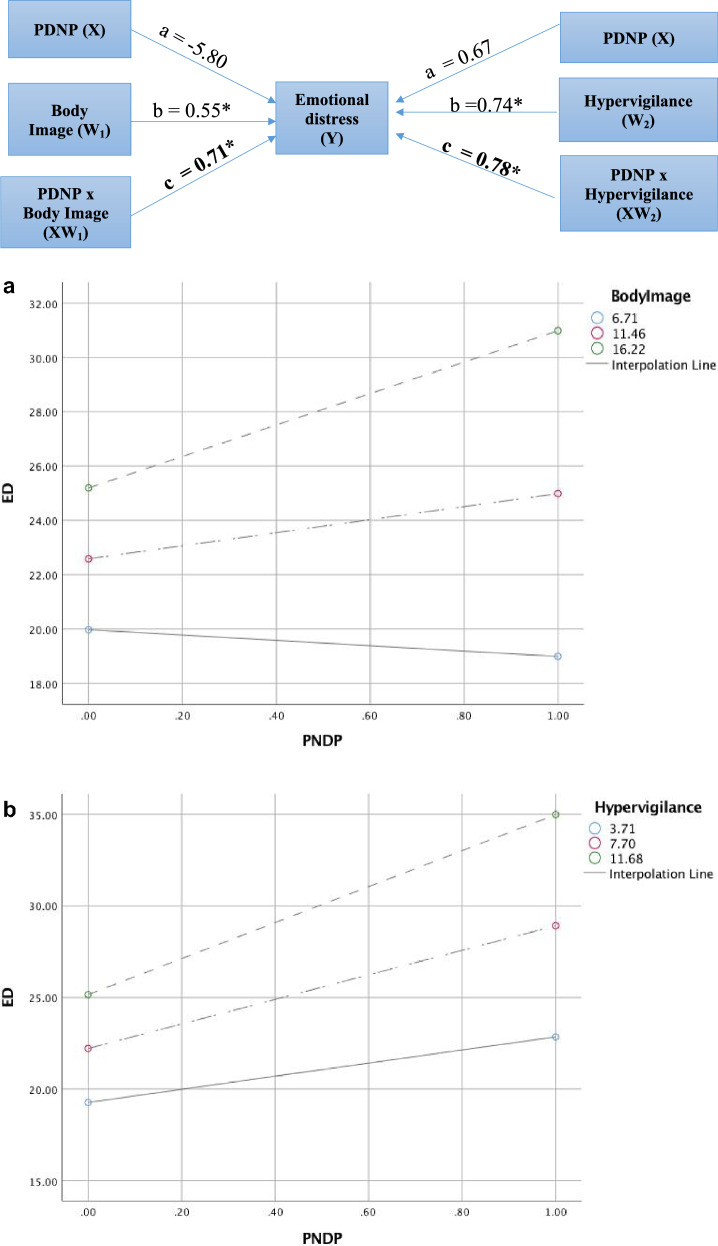
Moderating Effect of body image and hypervigilance on Emotional Distress. Figure 2a. Conditional effects of PNDP on Emotional distress at values body image. Figure 2b. Conditional effects of PNDP on Emotional distress at values of hypervigilance

The conditional effects of PNDP on ED were significantly different at the three levels of body image. Among the ALPHIV who had body image scores one SD higher than the mean, the relationship between PNDP and ED was statistically significant (5.80, 95% CI: 1.87 – 9.71). However, the relationship between PNDP and ED at the mean score (2.40, 95% CI: -1.53 – 6.33) and one SD below the mean (-0.98, 95% CI: -6.85 – 4.90), were not statistically significant (Fig. 2a).

Parental control and assuming ALPHIV to be asexual being had led to the emergence of ED in the qualitative phase. Hypervigilance emerged as a significant moderator for the relationship between PNDP and ED (Table 4).The conditional effects of PNDP on ED were significantly different at three levels of hypervigilance (Fig. 2b).

For the ALPHIV who reported hypervigilance one SD more than the mean (9.82, 95% CI 5.59–14.05) and at the mean (6.70, 95% CI 3.40–10.00), the relationship was significant. However, the effect of PNDP on emotional distress at hypervigilance score one SD below the mean (3.58, 95% CI − 0.98–8.14) was not significant. Therefore, among ALPHIV with mean and greater than mean levels of *hypervigilance* from parents/guardians, the effect of PNDP on ED was significantly stronger.

**Fig. 3:**
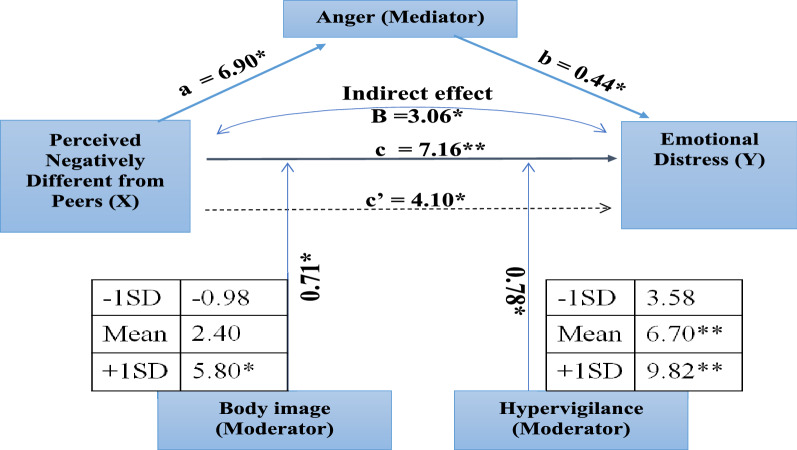
Model showing pathways leading to Emotional Distress among ALPHIV

Anger was not found to be statistically significant as a moderator (PNDP x Anger, Path c, B = 0.32, p = 0.093) for the relationship between PNDP and emotional distress. Therefore, the final emerging model shows that anger explains the mediating mechanism while body image and hypervigilance have moderating effects on the relationship between PNDP and emotional distress (Fig. 3).

## Discussion

This study provides an insight into the impact of HIV on the emotions of ALPHIV who acquired HIV vertically and therefore, were clueless about risk behaviors. They shared their innermost thoughts which revealed their foremost anxieties about: (1) becoming friendless, (2) being different from others, (3) thrown out of school, (4) challenges about marriage & compulsive abstinence expected of them, and (5) not achieving future goals in terms of career. Thoughts about source of infection made them wonder about their parents. Protection from stigma bordered to such hypervigilance that it rendered ALPHIV to self-imposed or parent-imposed isolation which led to expectation of not moving towards building friendship and/or sexual relationship. ALPHIV seemed to compel to believe that marriage /sexual relationship is ‘not for them’. However, the very nature of adolescence; the growth and development promulgates these needs naturally which in ALPHIV gets suppressed. Our study indicates the suppressed desire that could lead to syndemic of unfulfilled sexual desires, emotional distress, and career failures.

The constricted environment gives rise to emotional distress (ED). Through the qualitative exploration, various emerging stressors such as PNDP, anger, body image, and hypervigilance which were confirmed through mediation and moderation analyses. These stressors led to ED among ALPHIV. Appearance or report of these stressors are critical point of intervention. Among the study participants, anger was an easily identifiable and visible emotion; ALPHIVs were vocal about body image issues and parental hypervigilance. In contrast, ED is difficult to express and decipher as it gets masked by the physical complaints which are explained “understandable” distress of the disease [15, 16].

ED is considered a precursor to mental health disorders and if unaddressed, it might take short time to reach the level of depression. Studies have shown that ALPHIV is at higher risk of ED and other mental health disorders like depression, anxiety, and stress disorders [1, 9, 19]. Our study has shown high ED among ALPHIV and it is critical to address ED because there are no guidelines for its assessment. Anger was found to be explaining the mediation pathway between PNDP and ED while body image and hypervigilance had moderating effects, similar to a study among Iranian students that reported the mediating role of anger rumination on the relationship between anger and psychological well-being [33]. ALPHIV had several reasons for being angry such as getting a disease from a parent, a chronic disease requiring lifelong treatment, and feeling of being very different from their peers. One plausible explanation for how anger could function as a mediator can be explained using Bowlby’s Attachment theory (1973) [29]. Among ALPHIV, healthy child development alters by HIV infection at the family, individual, and societal/community level, leading to insecure attachment with parent/caregiver, peers, and even with an intimate partner [34, 35]. Healthy relationship establishes a sense of security while disturbed relationships, with repeated threats of rejection from an attachment figure, leads to the development of distorted emotional responses such as anger [29]. Knowing one’s own HIV status or confusion about “what is wrong with me”, and “why me” leads to the expression of anger among ALPHIV towards their parents thus leading to less adherence [36]. Since anger can be identified easily, anger assessment and management are recommended as part of adolescent counseling for the primary prevention of ED.

Worldwide, healthcare providers emphasize that ALPHIV or any HIV infected adolescent should live “normal” lives similar to their uninfected counterparts. However, several studies have shown that all types of ALHIV reported feeling “different” from others or their peers because of the presence of HIV and the need to take daily medications as compared to their peers [11, 37, 38]. This evidence supported the findings of our study where the ALPHIV reported feelings of “being different” from their peers. Adolescents’ self-perception of their own social identity and positive internal sense of social acceptance is associated with their psychosocial well-being [39].The inherent stigma of HIV results in a negative sense of self among ALPHIV, leading to feeling “different” from others and social withdrawal from peer groups and social activities. For example, Petersen et al. reported that 36% of adolescents withdrew from their friends and social activities on finding out their HIV-positive identity [37].Therefore, negative perception about oneself leads to ED among ALHIV.

Body image, an individual's perception of one’s own body, is a significant aspect of identity formation among adolescents. Beliefs and perceptions about body image when does not fit with societal standards, it impact the psychological well-being of the adolescent resulting in high ED [40], which gets highlighted in our study. Other studies have also reported PNDP, body image, and hypervigilance as predictors for the development of ED [33, 38, 40, 41]. We found that the relationship between PNDP and ED was moderated by the function of body image. Therefore, for an ALPHIV with a negative body image, the effect of PNDP on ED was significantly stronger as compared to ALPHIV with a positive body image. In the latter, the effect of PNDP on ED was non-significant. Other studies have shown that quality of life was affected among those HIV-infected individuals who reported lipodystrophy as they were on antiretroviral therapy for a long time [42, 43]. PNDP emerged as a common circumstance in the African ALHIV which revealed body image and mental health issues [44]. Thus, ALPHIV-specific counseling needs to focus on body image issues, provide correct knowledge about body shapes and sizes; help them accept their bodies, and inspire them to find beauty within.

Our study also identified parental issues that affected the emotional well-being of the ALPHIV. Controlling adolescents is a core conflict between parents and adolescents. Both ALPHIV and parents cited various instances of over-protectiveness or being hypervigilant for their HIV-infected ward/s. For example, parents/guardians put restrictions on going out with friends, school trips, and even on romantic/sexual relations among ALPHIV. Parents/guardians expected ALPHIV to abstain from sexual activities similar to other studies [45, 46]. The parent’s fears of secondary transmission of infection and lack of adolescence-specific developmental transitions culminate in control of ALPHIV’s sexuality. However, romantic/sexual relations influence the emotional well-being of adolescents. It is evident that in the HIV care cascade, the sexual needs of ALPHIV are unmet [47] and parents are also not counseled accordingly. Although communication on reproductive and sexual health issues with children/adolescents is considered taboo in India [48], our study shows that parents seemed to communicate about sexual relationships with their HIV-positive ward. Paradigm shifts are needed to provide effective health and social services to adolescents acknowledging in respectful and age-appropriate ways, these aspirations related to marriage/sexual relationship provide an opportunity to engage adolescents in integrated sexual and reproductive health (SRH) services, including HIV care and treatment services. Yibrehu et al. reported that sexual explorations by adolescents trigger parental communication on RSH and HIV [49]. Studies show that parental communication in sensitive areas especially sexuality is known to influence risk behavior among adolescents and is also associated with positive mental health outcomes among ALHIV [9, 50, 51]. Therefore, skill building of parents/guardians by increasing the knowledge about adolescence-specific developmental changes is recommended. The ALHIV and parents both need to be informed about the concept of positive prevention which may consist of the PPTCT program, the importance of ART adherence, maintaining a low viral load and high CD4 count, condom use for the protection of others, and assisted reproductive technologies such as sperm washing, intra-uterine insemination (IUI) and in-vitro fertilization (IVF) for safe sex practices and safe pregnancy.

The parental over-protectiveness or hypervigilant behaviour might explain the ED or anger among ALPHIV who do not want to be different from their peers and want to conform and participate in all activities like any other adolescent.

The need to measure parental hypervigilance and its impact on the emotional well-being of ALPHIV has emerged. Lee et al. reported that children who scored lower on their self-esteem and higher on stress levels reported having more overprotective parents [41]. It would be prudent to differentiate between parental protection or care or overprotection. A study from China recommended that because less parental care brought behavioral problems in ALHIV; parental and family-level factors should be considered when providing care and support to ALHIV [52]. However, overprotection or hypervigilance by the parents of ALHIV in India showed a moderating relationship between PNDP and ED. The effects of PNDP on ED were significantly different at varying levels of hypervigilance. The relationship was strongest when hypervigilance scored higher than the mean score. The direction of the relation between distress and parental overprotection is also debated by Bokszczanin who felt that parental overprotection may lead to distress among adolescents [30]. However, Hudson and Rapee felt that distress promotes parental overprotection [53]. Gillespie et al. highlighted the direction of causation between parenting behavior and ED and revealed a better-fit model which specified parental behavior as the cause of ED [31]. Sometimes it is the parent’s own stress that culminates in terms of overprotection which might be claustrophobic and inadvertently encourage timidity in a child in a highly developmental stage who is looking for self-identity and independence [54]. Our study brings out an important need of training parents of ALPHIV to understand the difference between ‘care’ and ‘over protection’. Culturally-tailored checklist of hypervigilance through the parents can be developed in the future. Our study provides the evidence to plan and design focused interventions to prevent ED among ALPHIV. Findings emphasize the need for counseling guidelines that include anger assessment and management, focus on body image issues, and being alert to the reports of hypervigilance by the parents.

### Study limitations

In the qualitative phase, we could not conduct focus group discussions (FGD) among ALPHIV due to confidentiality and psychosocial reasons. FGD of HCPs was also not conducted because of not getting an adequate number of HCPs who had experience in the management of ALHIV. The cross-sectional survey limits the inferences of the causality of the mediation analyses in this study. However, a longitudinal study design would be better equipped for conducting such an analysis. Although several studies have employed cross-sectional study designs for mediation analyses, the temporality and causality of predictors of ED in our study should be interpreted cautiously and the mediating relationship should be considered as an indirect association. Another limitation was we have not conducted power analyses a priori for this pilot exploratory study and due to the small sample size, this study may not have adequate statistical power to detect some of the statistically significant associations. The purposive sampling method employed in our study focusing on adolescents in HIV care, excluding orphan adolescents, those who acquired HIV behaviorally, and having any cognitive limitations, restricts the generalizability of our study findings to the broader sections of ALHIV in India. In addition, the data were collected using the self-reported measures of participants, this could be influenced by potential subjective bias.

## Conclusion

Anger mitigation, addressing body image issues and self-perception emerge as critical components of ALPHIV counseling. Anger assessment and management, body image and identity formation issues, and being conscious of the reports of hypervigilance by parents need to be addressed for the primary prevention of emotional distress among ALPHIV. Parents/guardians need to change attitudes and introspect about their own fears so that they may avoid hypervigilant behaviors. Programmers and parents need to be cognizant of developmental changes in the ALPHIV and ensuing sexual desires which need appropriate counselling and acceptance.

## Data Availability

Data will be available at the ICMR-NARI’s centralized data repository system and is available on request to director@nariindia.org.

## Abbreviations

ALHIV: Adolescents Living with HIV
ED: Emotional Distress
PNDP: Perceived Negatively Different From Peers
ART: Antiretroviral Therapy
IDI: In-Depth Interviews
KII: Key Informant Interviews
NGOs: Non-Government Organizations
HCPs: Healthcare Providers
ICTC: Integrated Counseling and Testing Centre
APR: Adolescent-Parent Relationships
APC: Adolescent-Parent Communication
RSH: Reproductive and Sexual Health
SD: Standard Deviation
FGD: Focus Group Discussion
